# Transport of neutral IgG2 versus anionic IgG4 in PD: implications on the electrokinetic model

**DOI:** 10.1186/s12882-018-1104-1

**Published:** 2018-10-29

**Authors:** Anneleen Pletinck, Wim Van Biesen, Clement Dequidt, Sunny Eloot

**Affiliations:** Nephrology Division, Ghent University Hospital, C. Heymanslaan 10, 9000 Ghent, Belgium

**Keywords:** Peritoneal Dialysis, Transperitoneal membrane transport, Immunoglobulin, Three pore theory, Elektrokinetic model

## Abstract

**Background:**

It is debated whether transperitoneal membrane transport of larger (charged) molecules in peritoneal dialysis can be partially governed by the electrokinetic model. In this model, it is postulated that streaming potentials are generated across the capillary wall by forced filtration of an ionic solution, for example transcapillary ultrafiltration induced by osmotic forces as in peritoneal dialysis. We investigated the presence of streaming potentials in the process of transperitoneal transport in Peritoneal Dialysis (PD) patients by measuring ratios of dialysate concentrations of IgG2 (neutral) and IgG4 (negative), both 150kD, under different conditions of transcapillary ultrafiltration.

**Methods:**

Adult PD patients randomly got two consecutive dwells of 120 min each, with either 2 L Physioneal 1.36% or 3.86% glucose dialysis fluid (Baxter, USA) as their first dwell. A blood sample was taken at the test start, and dialysate samples were taken at 5, 15, 30, 60 and 120 min. IgG2 and IgG4 concentrations were measured (ELISA) and ratios calculated.

**Results:**

In 10 patients (65 ± 17 years, 20 ± 17 months on dialysis), drained volume after 120 min was different between the 1.36% (1950 [1910; 2020] mL) and 3.86% (2540 [2380; 2800] mL) glucose dwells (*P* = 0.007). At none of the time points and irrespective of glucose concentration, a significant difference was found between the IgG2/IgG4 ratios at any time point.

**Conclusion:**

Our data failed to demonstrate a difference in the transport ratios of two macromolecules with same molecular weight but different charge, as would be expected by the electrokinetic model, and this despite sufficient differences in transcapillary ultrafiltration.

**Clinical trial registry:**

Belgian Registration Number B670201523397 (20/1/2015); prospective randomized trial.

## Background

Within the electrokinetic model, streaming potentials are generated across a filter by forced filtration of an ionic solution [1]. The force and direction of the induced electrical field are in theory determined by the amount of flux through the filter pores, and add another transport force through electrophoresis, influencing the passage of charged macromolecules across the pores [2]. This hypothetical electrokinetic force was previously not considered to be present across capillary walls. It is usually observed that capillary walls are negatively charged, and the electrical field should thus be positive on the outside and negative on the inside of the capillary wall. As most plasma proteins (e.g. albumin) are negatively charged, the polarity of such electrical field would result in increased transcapillary transport. In reality, these negatively charged molecules appear to be repelled from the pores towards the capillary lumen, which would presume the presence of a reversed streaming potential [3]. Whereas this seems plausible from the theoretical perspective, and fits with observational data, its occurrence in real life is still a matter of debate. Recently, the presence of reversed streaming potentials was reported in the glomerular membrane of Necturus [4] and the bovine lens basement membrane [5].

In peritoneal dialysis (PD) it is accepted that solute transport across the peritoneal membrane can be modelled by the three pore model [6], where the transport barrier consists of a serial coupling of two distinct systems: the interendothelial slits of the capillary wall itself, and the matrix of the interstitial tissue in which the capillary is imbedded. This results in a much longer diffusion distance than for example in a human glomerulus [7]. As a consequence, transport over the peritoneal membrane behaves more like that in a gel column, whereas transport in the human glomerulus behaves more like that of a synthetic dialyser [8]. Available evidence seems to suggest that electrostatic forces have little impact in this system. There is apparently no electrostatic charge selectivity over the peritoneal membrane [9, 10]. It has been debated whether this transport can also be governed by electrokinetic forces [11–14]. In this model, it is postulated that streaming potentials are generated across the capillary wall by forced filtration of an ionic solution, for example transcapillary ultrafiltration induced by osmotic forces as in peritoneal dialysis. Accordingly, transport of solutes with the same molecular weight but different charge (e.g. IgG2 & IgG4) would be different at different time points during the dwell, an effect that would be further enhanced when transcapillary ultrafiltration is enhanced by using hypertonic glucose.

If this electrokinetic model exists in the peritoneal membrane, it will alter our understanding of transperitoneal transport, potentially opening new opportunities to use alternative osmotic agents, develop protective strategies, or detect early changes in peritoneal membrane integrity. Furthermore, a better understanding of streaming potentials would lead to a better insight in the increased transperitoneal protein loss in PD over time.

Our study therefore intends to investigate the hypothesis of the presence of (reversed) streaming potentials in the process of transperitoneal transport in PD patients by measuring dialysate IgG2/IgG4 concentration ratios, under different conditions of transcapillary ultrafiltration. These differences were induced by using non-hypertonic vs hypertonic glucose, and by measuring at different time points during the dwell, as transcapillary ultrafiltration is also decreasing with dwell time. If streaming potentials do play a role, these differences in transcapillary ultrafiltration will result in different IgG2/IgG4 ratios during the dwell, and also between the non-hypertonic and hypertonic exchanges.

## Methods

Consecutive adult patients on peritoneal dialysis in the Ghent University Hospital were asked consent for participation in this prospective randomized cross-over study until 10 patients had completed the study. Exclusion criteria were active infection, pregnancy, unstable hemodynamic condition precluding use of hypertonic glucose, peritonitis in the last 4 weeks preceding the study, and age below 18. Patients with malfunctioning catheters were excluded. Malfunctioning was assessed based on medical history. In addition, when on a routine Peritoneal Equilibration Test (PET) test residual volume was more than 15%, the patient was also excluded.

The study was approved by the local Ethics Committee (Commissie voor Medische Ethiek - UZ Gent - Ref 2015/0075 - Belgian Registration Number B670201523397), and written informed consent was obtained from all participants.

**Fig. 1:**
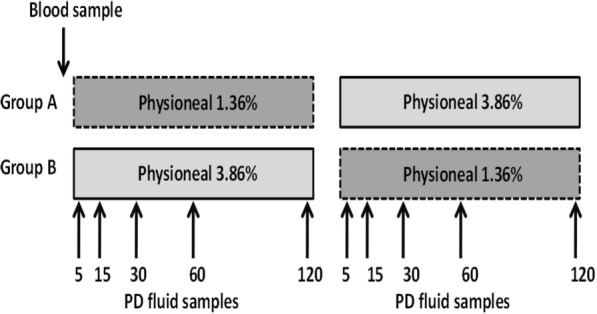
Study set up

Study flow is depicted in Fig. 1. Patients got two consecutive dwells of each 120 min with either 2 L Physioneal 1.36% or 2 L Physioneal 3.86% glucose dialysis fluid (Baxter, USA) as their first or second dwell. Randomisation was obtained with www.randomization.com.

A blood sample was taken from the patient before the test start, and dialysate samples were taken during each dwell at 5, 15, 30, 60 and 120 min via the peritoneal catheter. At each of these time points, the patient was first rolled from side to side to enhance optimal fluid mixing in the peritoneal cavity before 200 mL dialysate fluid was drained to diminish the impact of remaining fluid in the catheter’s dead volume. Of this sample, 30 mL was sampled and put on ice for transport to the laboratory, while the remaining 170 mL were re-instilled into the peritoneal cavity. After 120 min dwell time, dialysate fluid was drained completely, and the volume checked using gravity.

Samples were immediately centrifuged at 4 °C during 10 min (3000 rpm for blood samples, and 1800 rpm for dialysate samples), after which the serum and dialysate were stored at − 80 °C until batch analysis. For all samples, IgG2 and IgG4 concentrations were estimated using commercially available ELISAs (platinum ELISA, eBioscience, USA). Preliminary tests were performed to check the most optimal dilution of the samples for the respective ELISAs; for serum, dilutions were needed of 1/500,000 (IgG2) and 1/5000 (IgG4), and for dialysate fluid dilutions were 1/500 (IgG2) and 1/10 (IgG4 at time point 5, 15, and 30 min) and 1/50 (IgG4 at time point 60 and 120 min).

For each time point and in each patient, the ratio of IgG2 over IgG4 concentrations was calculated.

### Specificity

The IgG2 and IgG4 assays detect both natural and recombinant human IgG2 and IgG4 respectively. No cross reactivity or interference of circulating factors of the immune system was detected. The curves obtained by serial dilutions of the dialysate was parallel to the standard curve.

### Sensitivity

The limit of detection of resp. human IgG2 and IgG4 defined as the analyte concentration resulting in an absorbance significantly higher than that of the dilution medium (mean plus 2 standard deviations) was determined to be resp. 0.25 ng/ml and 0.1 ng/ml (mean of 4 independent assays).

### Precision

The intra-assay coefficient of variation for serum samples was 3.6% for IgG2 and IgG4. The intra-assay coefficient of variation for dialysate samples was 4.3% for IgG2 and 5.9% for IgG4.

### Statistical methods

Statistical analyses were performed with SPSS 23 (IBM©). To compare results from the 1.36% to 3.86% dwell, patients were considered their own control, so a paired non-parametric analysis was applied (Wilcoxon Signed Rank test for non-normally distributed data). For the evolution of IgG2/IgG4 over the dwell time, a repeated measures Friedman analysis was applied. We also constructed a general linear model for ratio of IgG2/IgG4, including patient identification, osmotic tonicity and time points as covariates, and interaction terms for tonicity and time points.

No formal sample size calculation was performed, as this was deemed not reliable as no data were available to estimate effect size or standard deviation and patients served as their own controls.

## Results

Ten patients (2 women; 4 with diabetes mellitus), 65 ± 17 years of age, 20 ± 17 months on dialysis, and with a residual renal function of 9.7 ± 5.6 mL/min/1.73m^2^ at the time of the study were included. Drained volume after 120 min was different between the 1.36% and the 3.86% glucose dwells: i.e. 1950 [1910; 2020] mL versus 2540 [2380;2800] mL, respectively (*P* = 0.007).

Serum concentrations were 1.7 [0.8; 2.9] mg/mL for IgG2 and 1.3 [0.2;2.4] mg/mL for IgG4.

**Fig. 2:**
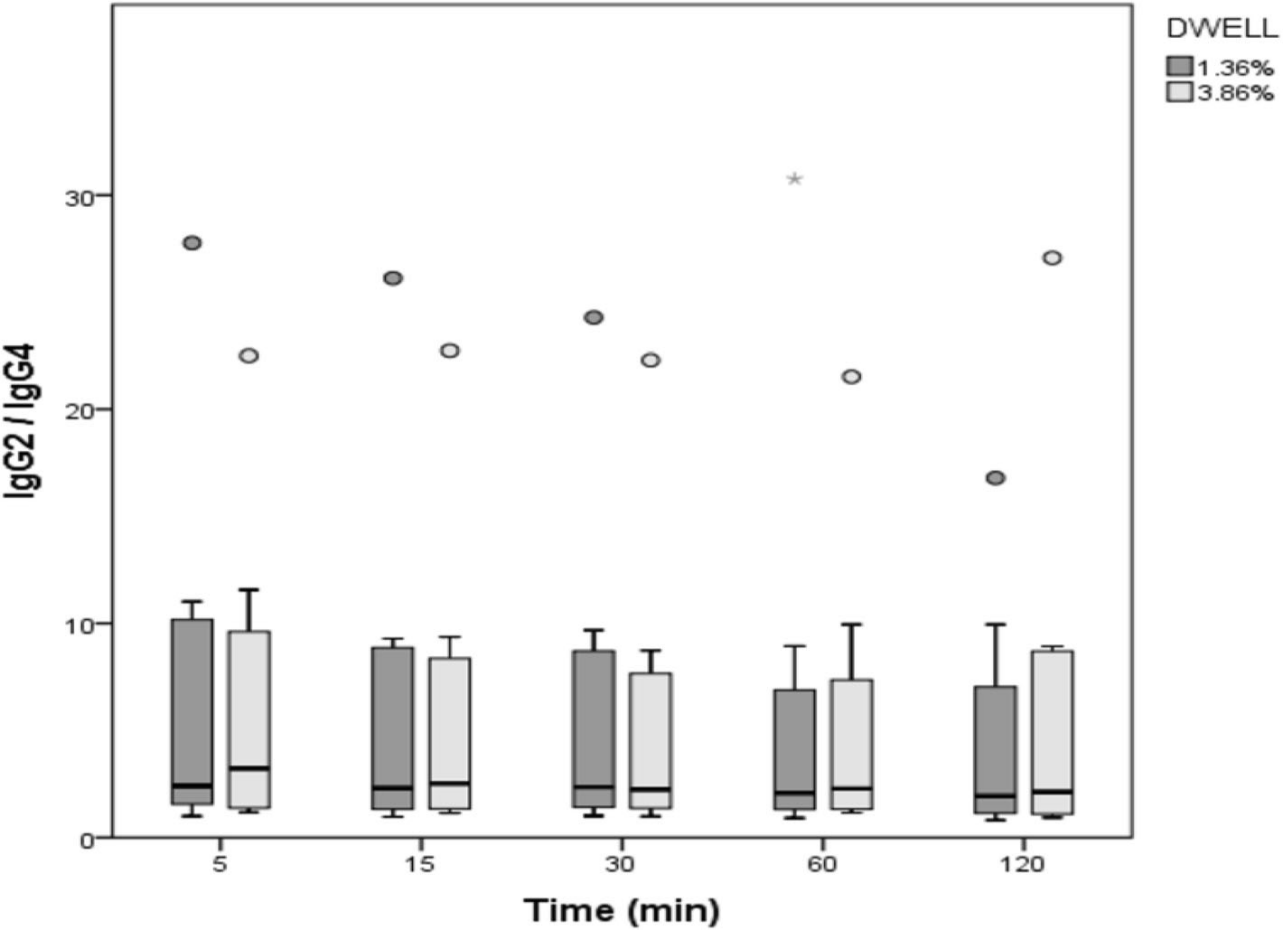
IgG2/IgG4 ratio at different time points during the PD test session for dwells with Physioneal 1.36% (dark bars) and Physioneal 3.86% (light bars)

IgG2/IgG4 concentration ratios in dialysate are shown in Table 1 and Fig. 2 for the different time points during the PD test sessions, and for the different used PD fluids, i.e. Physioneal 1.36% versus 3.86%. At none of the time points, a significant difference was found between the 1.36% and the 3.86% ratios (Table 1). In none of the patients a difference in IgG2/IgG4 ratio was found at different time points of the dwell, neither when using the 1.36 nor the 3.86% solution.

**Table 1:** IgG2/IgG4 ratios at different time points during the PD test session with Physioneal 1.36% and Physioneal 3.86%

Time (min)	Physioneal 1.36%	Physioneal 3.86%	*P*-value
5	2.4 [1.5; 10.6]	3.2 [1.3; 10.6]	1.00
15	2.3 [1.3; 9.1]	2.5 [1.2; 8.9]	1.00
30	2.3 [1.4; 9.2]	2.2 [1.3; 8.2]	0.13
60	2.1 [1.2; 7.9]	2.3 [1.3; 8.7]	1.00
120	1.9 [1.1; 8.5]	2.1 [1.1; 8.9]	0.73

Median [interquartile range]

In the general linear model (*p* < 0.001, R^2^ = 0.022,), only patient identification but not use of hypertonic vs non-hypertonic bags or timepoint of dwell had an impact on IgG2/IgG4 ratio (Table 2).

**Table 2:** General linear model for IgG2/IgG4 ratio

Source	Type III Sum of Squares	df	Mean Square	F	Sig.
Corrected Model	1040,462^a^	5	208,092	4620	,001
Intercept	6375	1	6375	,142	,708
Tonicity	7999	1	7999	,178	,675
Timepoint	7579	1	7579	,168	,683
Tonicity * timepoint	8309	1	8309	,184	,669
Patient identification	499,674	1	499,674	11,094	,001
timepoint * patient identification	2015	1	2015	,045	,833
Error	3603,201	80	45,040		
Total	8061,235	86			
Corrected Total	4643,663	85			

**Fig. 3:**
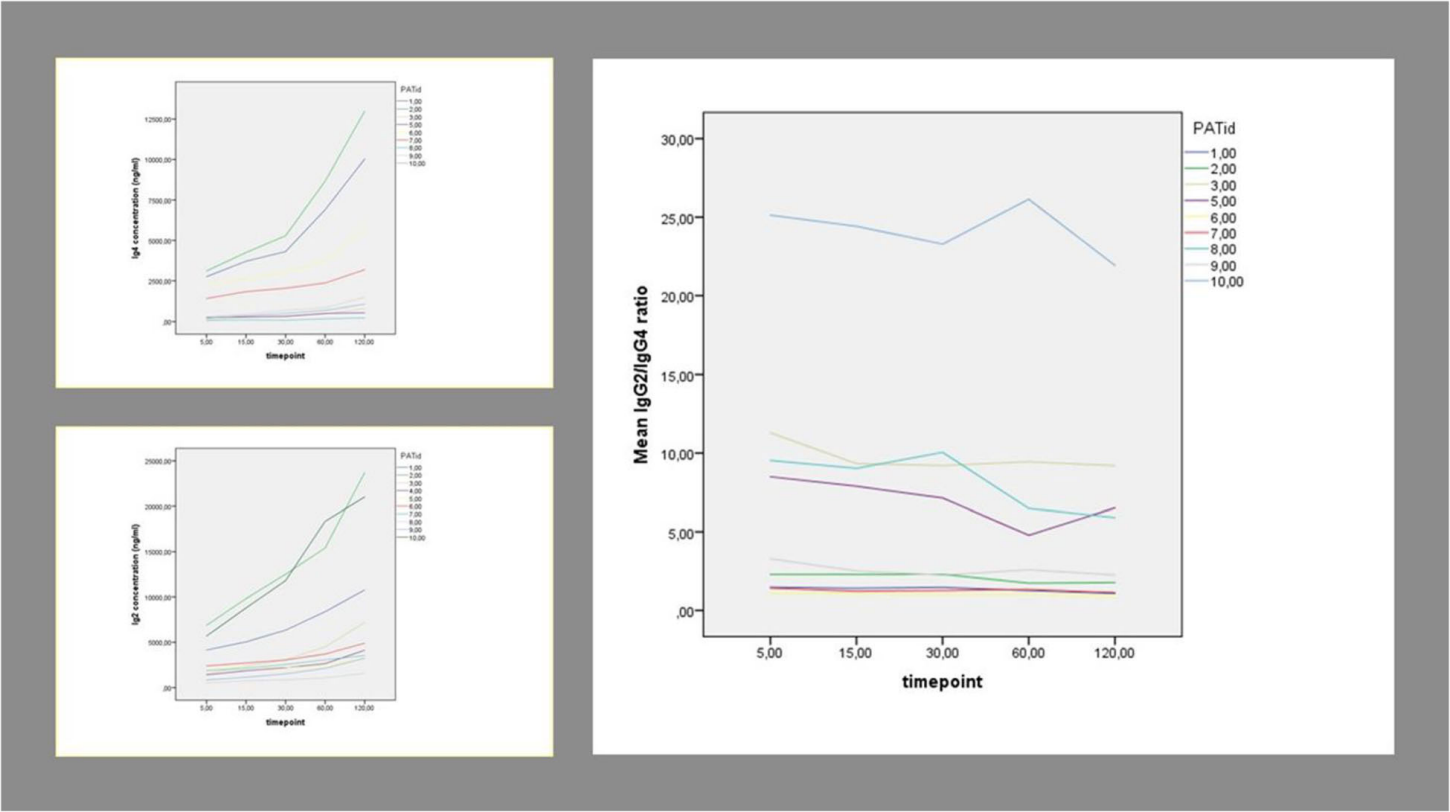
Evolution over time of IgG2 and IgG4 concentrations and the IgG2/IgG4 ratio in individual patients

Raw data for individual patients of concentrations of IgG2 and IgG4 in serum and in dialysate at different time points for the hypertonic and non-hypertonic exchanges are presented in Tables 3 and 4. Figure 3 represents the evolution during the dwell of IgG2, IgG4 and the IgG2/IgG4 ratio for individual patients.

**Table 3:** dialysate concentrations for IgG2 and IgG4 at different timepoints for the different glucose strengths

PatientN°	dwell	Time point	IgG2 (ng/ml)	IgG4 (ng/ml)	Ratio (IgG2/IgG4)
1	1,36%	5	5824,5	3772,9	1, 54
		15	6746,5	5239,7	1, 29
		30	8210,5	5573,6	1, 47
		60	11,730,5	10,122	1, 16
		120	14,743,5	13,771	1, 07
	3,86%	5	2455,9	1733,8	1, 42
		15	3344,1	2185,2	1, 53
		30	4464,6	3043,9	1, 47
		60	5014,5	3670,8	1, 37
		120	6808,0	6290	1, 08
2	1,36%	5	9411,0	4465	2, 11
		15	12,267,5	5136,1	2, 39
		30	16,719,5	6263,2	2, 67
		60	20,711,0	10,830,5	1, 91
		120	33,158,0	17,144,5	1, 93
	3,86%	5	4321,2	1745,9	2, 48
		15	7379,0	3371,3	2, 19
		30	8311,0	4307	1, 93
		60	10,110,5	6497	1, 56
		120	14,258,0	8826,5	1, 62
3	1,36%	5	1028,6	93,335	11, 02
		15	1551,4	167	9, 29
		30	2284,8	236,06	9, 68
		60	3461,4	387,02	8, 94
		120	6653,0	668,75	9, 95
	3,86%	5	1994,5	172,41	11, 57
		15	3157,1	336,96	9, 37
		30	3868,1	442,99	8, 73
		60	5520,0	554,5	9, 95
		120	7742,5	916,15	8, 45
4	1,36%	5	870,5	/	/
		15	1294,4	/	/
		30	1732,4	/	/
		60	2317,0	/	/
		120	3979,2	/	/
	3,86%	5	1933,7	/	/
		15	2425,8	/	/
		30	2663,9	/	/
		60	2977,1	/	/
		120	4251,7	/	/
5	1,36%	5	1243,1	133,23	9, 33
		15	1454,8	172,31	8, 44
		30	1701,4	220,51	7, 72
		60	1995,6	414,62	4, 81
		120	2706,5	658,97	4, 11
	3,86%	5	2793,9	365,08	7, 65
		15	3064,0	417,25	7,34
		30	2745,9	417,16	6, 58
		60	2813,2	591,48	4, 76
		120	3832,0	429,14	8, 93
6	1,36%	5	2322,8	2347,4	0, 99
		15	2703,9	2817,2	0, 96
		30	3528,3	3515,1	1, 00
		60	4134,3	4605	0, 90
		120	5747,5	7067	0, 81
	3,86%	5	2455,5	2125,6	1, 16
		15	2721,1	2423,5	1, 12
		30	2563,9	2614	0, 98
		60	3268,3	2860,65	1, 14
		120	4040,1	4378,95	0, 92
7	1,36%	5	672,1	435,84	1, 54
		15	878,6	668,1	1, 32
		30	1158,8	883,53	1, 31
		60	1793,4	1257,8	1, 43
		120	2564,0	2202,15	1, 16
	3,86%	5	3099,6	2373	1, 31
		15	3363,4	2987,7	1, 13
		30	3941,3	3195,9	1, 23
		60	4324,0	3469,4	1, 25
		120	4521,3	4169,65	1, 08
8	1,36%	5	374,6	/	#WAARDE!
		15	609,5	/	#WAARDE!
		30	758,1	85,828	8, 83
		60	1071,6	/	#WAARDE!
		120	1803,2	/	#WAARDE!
	3,86%	5	619,2	65,01	9, 52
		15	905,7	100,245	9, 03
		30	966,7	85,828	11, 26
		60	1071,6	165,245	6, 48
		120	1367,3	231,85	5, 90
9	1,36%	5	1027,6	387,02	2, 66
		15	1303,1	591,82	2, 20
		30	1702,0	851,32	2, 00
		60	2204,0	1002,75	2, 20
		120	3261,2	1710	1, 91
	3,86%	5	665,5	169,33	3, 93
		15	979,5	347,67	2, 82
		30	1337,8	533,77	2, 51
		60	2037,2	686,15	2, 97
		120	3241,4	1244,8	2, 60
10	1,36%	5	7565,5	272,53	27 ,76
		15	10,973,5	420,25	26, 11
		30	14,616,0	602,27	24, 27
		60	24,084,0	783	30, 76
		120	24,346,3	1451,15	16, 78
	3,86%	5	3807,8	169,33	22, 49
		15	6596,5	290,23	22, 73
		30	8999,0	403,69	22, 29
		60	12,620,5	586,42	21, 52
		120	17,677,5	653,25	27, 06

**Table 4:** Serum values for IgG2 and IgG4 for individual patients at time point 0

PatientN°	IgG2 (ng/ml)	IgG4 (ng/ml)
1	1,758,200	2,379,000
2	3,954,600	2,490,400
3	1,053,150	173,835
4	875,000	/
5	256,640	84,665
6	1,810,300	3,030,500
7	831,750	1,284,000
8	686,250	150,925
9	1,656,800	1,917,900
10	7,310,500	764,950

## Discussion

This paper evaluates the plausibility that transperitoneal transport of macromolecules is governed by an electrokinetic force induced by reversed streaming potentials. In our experiments we failed to demonstrate a difference in the transport ratios of two macromolecules with the same molecular weight but with a different charge (neutral IgG2 and negatively charged IgG4), as would be expected if such electrokinetic forces would be present, and this despite the fact that sufficient differences were obtained in transcapillary ultrafiltration by using two different osmotic strengths of glucose. Also, no difference could be observed between the transport ratios at different time points, in contrast to what would be expected if the electrokinetic model would apply, as transcapillary ultrafiltration decreases during the dwell.

The electrokinetic model of transcapillary transport has been a subject of debate for a long time [11–14]. Proponents of the model claim that the model is able to explain several observations that cannot be explained otherwise by other models [3]. The pathophysiology of proteinuria for example is so far not completely explained by one of the existing models. Most strikingly, it is difficult to explain why proteins are not clogging up the pores of the glomerulus; existence of reverse streaming potentials would provide a sufficient explanation for this [1, 2]. So far, the presence of reverse streaming potentials over the glomerular basement membrane has only been demonstrated in mudpuppy (Necturus) [4]. However, it has been argued that the anatomical structure of the glomerulus of Necturus is much different from that in humans, and that as such it is difficult to make extrapolations to the human situation. The glomerular filtration barrier in Necturus is 10 fold thicker than in humans (3.5 μm vs 0.3 μm), and has a more “tissue like” appearance [12]. Also in the bovine lens basement membrane, the other setting where streaming potentials have been observed, the filtration barrier is much thicker, and has a tissue like structure.

In peritoneal dialysis, there is an observation of higher transperitoneal protein transport in conditions of inflammation [15, 16]. Whereas it is tempting to explain this by an increased recruitment of capillaries, and thus higher availability of large pores, adjusting for surface area increase does not completely abolish this effect, suggesting that inflammation alters large pore transport per se [17]. A change in electrical charge of the capillary wall, and thus in the streaming potential over the large pores, is an attractive alternative explanation. Presence of an electrokinetic model and streaming potentials could easily explain why transperitoneal leakage only occurs to a limited extent at one and, more substantially, at another moment, as the strength and direction of the electrical field would determine protein flux. It is important that this process, though also based on electrical charges, is different from pure charge selectivity, in which charged molecules cannot pass through a pore because its effective size is smaller than its actual (anatomical) size. Previous literature has demonstrated that such a charge selectivity cannot be observed in peritoneal dialysis [9]. It can be hypothesized that small and large pores are actually the same anatomical entity, but that differences in integrity of the glycocalyx or the interstitial space will govern transport properties by differing streaming potentials. Our results however do not add credibility to the hypothesis of streaming potentials being valid in peritoneal dialysis, as no differences were observed in the kinetic behaviour of IgG2 and IgG4, and this despite sufficient differences in generated flux by using different osmolarities of dialysate, and sampling at different time points in the dwell.

Different explanations can be forwarded to explain our negative findings.

First, it might be that IgG2 and IgG4 are not suitable to test the (hypothetical) impact of the electrokinetic forces in peritoneal dialysis. Although they have a different charge, IgG2 and IgG4 both have a comparable molecular weight around 150kD, which is 3 fold higher than that of albumin. Also, albumin has a globular structure, whereas immunoglobulins have not, but can have different, more tube-like shapes. As such, the hindrance in transport purely based on sterical hindrance can be so big that eventual small charge effects as would be induced by the electrokinetic forces in the peritoneal capillary, are overruled. However, at the level of the glomerular basement membrane, IgG2/IgG4 ratio was found to be decreased in patients with glomerulonephritis as compared to healthy volunteers (nearly 3 fold) or patients with other causes of underlying kidney disease [18]. This finding was attributed to a loss of charge selectivity of the glomerular barrier due to local inflammation. IgG2/IgG4 ratios have also been used to assess glomerular charge selectivity in non-diabetic renal disease, and correlate well with albuminuria. The large pores of the glomerular basement membrane are however much smaller (80–100 Ångstrøm) than the large pores of the peritoneal membrane (200–300 Ångstrøm), so that sterical hindrance is thus less likely to be an explanation for our observations. Potentially, other pairs of endogenous or exogenous molecules should be used to test the hypothesis that the electrokinetic model is valid in peritoneal dialysis, preferentially with different ranges of molecular weight and difference in charge.

Second, it might be that the electrokinetic model cannot be applicable to peritoneal membrane transport because of huge differences between anatomical structures surrounding the capillaries of the glomerular barrier versus those of the peritoneal membrane [8]. Whereas the glomerular barrier is very thin, and allows selectivity based on size, flexibility, shape and charge, the peritoneal membrane resembles more a gel filtration model. Solute transport pathways comprise the inter-endothelial slits, coupled in series with the interstitial space, which is much larger in the peritoneal membrane than in the glomerular basement membrane. Accordingly, again, eventual small effects induced by electrokinetic forces can be masked by bigger sterical hindrance for larger molecules. Reversal of streaming potentials has however so far only been reported in thicker membranes, such as bovine lens and the glomerulus of the Necturus, which have also a distinct anatomical configuration with a more expressed interstitial space, such as present in the peritoneal membrane.

Third, it might be that we did not achieve a sufficient gradient of solute drag and transcapillary ultrafiltration. However, we actually achieved a difference in ultrafiltration of 500 mL over a 2 h dwell, which is equivalent to a mean transcapillary ultrafiltration of around 4 mL/min. As transcapillary ultrafiltration is maximal at the beginning of the dwell, and decreases with duration of the dwell, the achieved values in the early stage of the dwell should be substantially higher than at the end. However, no difference in IgG2/IgG4 ratios at different time points was observed, neither with 1.36 or 3.86% glucose solutions.

Last, our set up might lack sufficient power to detect a meaningful difference, and sensitivity of the approach might be too low to detect a meaningful effect, especially as many other interfering forces might be at play in the clinical setting. Also Imholz et al. [19] could not find a difference in transport of larger molecules such as albumin, transferrin, IgG, IgA or alpha 2 macroglobulin after a dwell with an hypertonic vs non hypertonic glucose solution. However, in this experiment, analysis was only done after a 4 h dwell, so that any potential difference induced by streaming potentials would have been overwhelmed by cumulative diffusive transport. In addition, after 4 h, a difference in osmotically induced transcapillary ultrafiltration is unlikely to be still present, and, accordingly, no evaluation of a potential presence or absence of streaming potentials could be reasonably made in that setting. Additional experiments, using different indicator molecules, different ways to enhance convective solute drag, and larger patient groups or maybe animal models should be explored.

## Conclusion

Although the electrokinetic hypothesis is appealing to help explain transcapillary transport in peritoneal dialysis, we failed to provide evidence for the existence of streaming potentials in peritoneal dialysis. More sophisticated exploration of this intriguing hypothesis is warranted.

## Abbreviations

IgG2 and IgG4: Immunoglobulin 2 and Immunoglubulin 4
PD: Peritoneal Dialysis
PET: Peritoneal Equilibration Test
